# The development of a classification system for the treatment of scoliosis by the side shift

**DOI:** 10.1186/1748-7161-9-S1-O66

**Published:** 2014-12-04

**Authors:** Tony Betts

**Affiliations:** 1 Royal National Orthopaedic Hospital, London, United Kingdom

## Background

The Side Shift approach to correction of scoliosis curves has been used by therapist at the RNOHT for over 30 years. The Side Shift approach was developed by Mrs Min Mehta, and has been Modified using consensus based evidence of SOSORT. Clinical observations had indicated that not all patients could actively (Auto) correct to beyond the trunk midline, a key principle of Side Shift.

At the RNOHT a classification system based upon the ability of an individual to auto-correct the spine during a Side Shift movement has been developed to aid the appropriate application of the shift exercises and allow future comparative analysis.

## Aim

To develop a Clinical Classification System for the Physical Therapy treatment of scoliosis, which is reliable, valid and universally accepted.

## Methods

58 Consecutive patients who have AIS were tested, by two clinicians (a Physiotherapist and a Orthotist), in 2013. The clinicians were blinded to the classification of each other. The results were tested for reliability. Three types of Side-Shift were developed. Type 1: flexible, Type 2 :stiff, and type 3:rigid. Data was collected for comparison on hypermobility, Cobb angles,and ATR scores.

## Results

Agreement was measured using the Kappa statistic (κ).

### Intra-rater reliability

The kappa value for agreement between the raters measures on occasion one and occasion two showed substantial agreement, κ = 0.77, 95% CI (0.61 – 0.91), P < 0.01. There was good intra-rater reliability.

### Inter-rater reliability

The kappa value for agreement between the two raters measures showed substantial agreement, κ = 0.7623, 95% CI (0.504 - 1.000), P < 0.01. There was also good inter-rater reliability.

There was a moderate negative correlation between the Cobb angles and Hyperlaxity scores, r = -0.3847, p = 0.01.

Type 1 Side Shift accounted for 73% subject with an average Hypermobilty score of 6/9.

## Conclusion

The results suggest that the Side Shift classification is a reliable scale of descriptive mobility and ability to Auto-correct.
